# Editorial for the Special Issue: Environment Microorganisms and Their Enzymes with Biotechnological Application

**DOI:** 10.3390/microorganisms12010204

**Published:** 2024-01-19

**Authors:** Myung-Ji Seo

**Affiliations:** 1 Division of Bioengineering, Incheon National University, Incheon 22012, Republic of Korea; mjseo@inu.ac.kr; 2 Department of Bioengineering and Nano-Bioengineering, Graduate School of Incheon National University, Incheon 22012, Republic of Korea; 3 Research Center for Bio Materials & Process Development, Incheon National University, Incheon 22012, Republic of Korea

The ubiquitous nature of microorganisms demonstrates their ability to survive and thrive in diverse ecological settings, and their presence in extreme environments that approach the known limits of adaptable living confers importance to their role in those ecosystems. These feats drive our desire to understand microorganisms’ metabolites and how these compounds interact with the materials around them. Understanding this will facilitate the search for biomaterials that can be developed for the degradation of harmful substances, and the discovery of compounds with novel biotechnological features. Biological resources that can be utilized in various fields are identified through bioprospecting. The search for, collection and analysis of biological materials obtained from natural environments are conducted with the aim of unearthing novel compounds, genes or organisms with commercial or industrial applications. Furthermore, bioprospecting is growing indiscriminately, with exploration expanding from more common terrestrial ecosystems to biodiverse ecosystems with low or non-existent human populations, such as salt pans and lakes, sulfur pools, volcanoes, high altitude mountains, deserts, tundras and marine environments [1]. The metabolites and enzymes produced by microorganisms in these ecosystems could have impressive thermostability, cold adaptability, salt tolerance and resistance to environmental stressors that would not be tolerated by more common microorganisms [2]. Microbial enzymes are cost-effective, sustainable catalysts integral to biochemical and metabolic processes, and advances in genetic and metabolic engineering support the optimization of enzyme production and the ease with which their isolation and characterization are performed. Microbial enzymes from different ecosystems have diverse applications across various industries, including agriculture, cosmetics, textiles and pharmaceuticals [3]. 

Natural polymers have garnered significant attention due to their therapeutic potential. The exopolysaccharides (EPSs) of probiotic bacteria protect them from stressors and physical and chemical attacks [4]. EPSs are biodegradable and non-toxic, and can be found in microorganisms as either tightly bound capsules or loosely attached slime layers. The EPSs from *Lacticaseibacillus paracasei*, EPS DA-BACS, has been isolated from healthy human feces and found to exhibit gastrointestinal tolerance, anti-inflammatory and antifungal activity and antimicrobial activity against certain pathogenic bacteria [5]. Living lactic acid bacteria (LAB), as probiotics, confer health benefits to humans via the improvement or restoration of the gut microbiota after their consumption, and *L. paracasei* EPS DA-BACS can provide this through its loosely cell-bound EPSs. Notably, the glucomannan-type EPSs in *L*. *paracasei* EPS DA-BACS differs from other *Lactobacillus* species in its structural composition. Its EPSs support its high tolerance to acids, bile salts and pancreatin, promoting cell survival in gastrointestinal conditions. In addition, it possesses the prebiotic properties of promoting LAB gut colonization and inhibiting pathogenic bacteria through competition. Following the inactivation of the microorganism, or the removal of its EPSs via centrifugation, the EPSs can impart the aforementioned favorable health benefits to the human gut and also be used as a postbiotic. 

Endophytic fungi, the fungal communities residing within plant tissues, have a structural diversity and complexity to their secondary metabolites which make them an attractive reservoir for the discovery of new bioactive compounds. The cost-effectiveness and simplicity of the culture medium for fungal growth and metabolism have contributed to the heightened attention given to these cells. Endophytic fungi aid in protection and survival through their antimicrobial, antiparasitic, immunosuppressive, immunomodulatory, and biocontrol capabilities against plant pathogens. These fungi also facilitate nutrient assimilation and growth-promoting metabolites. García-Latorre et al. [6] evaluated the production of hydrolytic enzymes from fungi isolated from plants growing in the Spanish dehesas, grasslands with low, irregular rainfall, low soil fertility and eventual salinity. *Sarocladium terricola*, *Acremonium implicatum*, *Microdiplodia hawaiiensis* and an unidentified species were considered suitable for culturing with salt-containing residues or by-products of the agrifood industry. The eleven strains of fungi investigated mostly produced lipases and, while growth was variable during saline treatments, all strains were able to grow. Enzyme production was observed under both standard and salt-amended conditions; amylase production was not affected by the salt concentration and the majority of the strains with lipolytic activity still maintained enzyme production in saline media.

As a large percentage of the Earth’s surface is covered with water, the marine microorganisms within these bodies of water present opportunities for the discovery of novel metabolic pathways, enzymes and biomaterials. Bioactive molecules with antimicrobial properties against terrestrial pathogens are of particular interest because of pathogens’ growing resistance to current antibiotics. With a view to identifying novel compounds with pharmaceutical and industrial potential, Ruginescu et al. [7] investigated the diversity of marine bacteria in the Black Sea. The phylum Pseudomonadota was the most abundant of the taxa, and the main class of bacteria was Gammaproteobacteria, while the more common classes typically found in marine settings formed less than 2% of the community. The majority of the Black Sea strains exhibited a halophilic nature with a minimum requirement of 2.9% salts for growth. Some strains which did not require salts still showed extreme tolerance in up to 15% salt water. While the growth temperature was variable, a large fraction of the strains was able to grow at 4–40 °C, albeit slower at lower temperatures. The majority of the screened strains had at least one hydrolytic activity, with observed class dependence for hydrolytic enzyme production, as seen with Gammaproteobacteria generally producing lipases. Antimicrobial screening was performed against human pathogens and clinical isolates, and two strains from the *Aquimarina* and *Streptomyces* genera had antimicrobial activity against pathogenic microorganisms. Marine bacteria with high salt tolerance, cold adaptability and extracellular hydrolytic enzymes are relevant to various catalytic conditions in industrial applications and are advantageous over the currently used microorganisms and their enzymes.

Another marine environment-based study isolated the novel bacterial strain *Paenibacillus aurantius* MBLB1776T from a Korean marine environment. The strain was found to harbor pigments that impart an orange hue to cells and it is notably the first carotenoid-producing bacteria reported from the *Paenibacillus* species. Hwang et al. [8] studied the taxonomy and biodiversity of the *Paenibacillus* genus within marine mud environments, focusing on the isolated strain in order to explore its biotechnological potential. There is a distinct difference between the MBLB1776T strain and known strains within the *Paenibacillus* genus. The strain was described as a Gram positive, non-motile, spore-forming facultative anaerobe with resistance to only a few antibiotics. The strain contained the *crt* genes necessary for the formation of the carotenoid backbone and the genes present in the biosynthetic route of C_30_ carotenoids. It is expected that this strain should be able to synthesize a glucosyl or acyl C_30_ carotenoid with beneficial properties that can be exploited in the agricultural, pharmaceutical and cosmetic industries. 

Gene modification is one of the means of exploiting the data obtained from bioprospecting. In the industrial-scale production of enzymes or bacterial metabolites, stressors can limit the performance of microbial cell factories and limit the yields of desired products. The heterologous expression of genes capable of improving stress tolerance can be utilized for *Escherichia coli* hosts. Yang et al. [9] studied the resistance potential of *Deinococcus proteolyticus* to oxidative stress, UV-C stress and γ-radiation compared to *D. radiodurans*, which most research tends to focus on. *D. proteolyticus* exhibited greater oxidation tolerance and UV-C resistance than *D. radiodurans*, with a similar endurance to radiation stress. The gene (deipr_0871) encoding the response regulator had improved growth and oxidative stress resistance when expressed in *E. coli*, and enhanced poly-3-hydroxybutyrate (PHB) production. Manipulating regulatory factors, cell membranes and biosynthetic pathways are all means of improving the production capabilities of industrial strains of microorganisms.

Bioremediation encompasses processes that use plants, fungi or bacteria to degrade, remove or alter environmental contaminants and reduce their damage. When biodegradation processes occur with organic contaminants, they are typically converted into inorganic compounds via mineralization. The bioremediation of inorganic contaminants occurs via the altering of their transport properties, such as through complexation, accumulation, adsorption and redox reactions. Bisphenol A (BPA)-degrading microorganisms are bioremediation agents used in the degradation of this endocrine-disrupting chemical. Enzymes capable of BPA degradation differ by genus and species, and it has been observed that *Bacillus* has greater BPA-degrading capabilities than LAB [10]. Bacteria with excellent BPA-degrading capabilities were screened and characterized for their application in food. BPA, which is a typical microplastic, is present in the environment and has been found in the human body, and even excreted in human milk. As such, the development of a BPA-degrading food product could offer bioremediation properties to the body. In the study in question, BPA removal was strain-dependent and was highest in *B. subtilis* P74. Although lower than in culture medium, BPA degradation by *B. subtilis* P74 was still significant when applied to a solid-state media of fermented soybeans. Acidic conditions reduced BPA solubility while alkaline conditions inhibited bacterial growth, both affecting the bioremediation process. As *Bacillus* is generally regarded as safe (GRAS) and can be used in fermented food, it can also be developed as a probiotic with BPA-degrading properties. 

Three bioremediating strains of *Streptomyces* were isolated from honeybee samples and co-cultivated for their keratinase production [11]. Chicken feathers, a keratin-containing waste product, were degraded by *Streptomyces griseoaurantiacus* AD2, *S. albidoflavus* AN1 and *S. drozdowiczii* AD1. *Streptomyces griseoaurantiacus* had the highest keratinolytic activity, with slight antimicrobial activity against *Staphylococcus aureus.* Chicken feathers were used as the sole nutrient source, and co-culturing the three strains led to increased growth, metabolite production, keratin degradation and antimicrobial activity. *Streptomyces* bacteria with keratinolytic activity can be used as biofertilizers, promoting plant growth and suppressing diseases in symbiosis with plants and other microorganisms. 

Gonzalez et al. [12] described the benefits and risks associated with soil thermophile activity through the lens of climate change. Soil thermophiles use adaptable extracellular enzymes to mineralize organic compounds and release inorganic nutrients, and can maintain their activity in environments with high temperatures and low water levels. The role of soil thermophiles in bioremediation is due to the nutrient recycling that allows them to take organic matter and release complexes of inorganic nutrients into that organic matter. While this is beneficial for soil quality and plant growth, the excessive activity of soil thermophiles during hot and dry periods can lead to nutrient reduction and increased carbon dioxide release due to the continual decomposition and extensive mineralization of organic matter. The increased activity of soil thermophiles can also encourage soil aridity when combined with increased radiation, heat and desiccation. 

The study of microorganisms from different ecosystems cannot solely focus on their beneficial properties and the means of exploiting their metabolites and enzymes. We also need to identify the negative impact of microorganisms on various branches of applied technology. Microorganisms affect the structures of construction products like thermal insulation and building materials, rendering it necessary to prevent their growth and colonization. Plant-based materials are susceptible to microbial degradation, which is exacerbated by environmental conditions like elevated humidity and temperature. Seed oils have been proposed as alternatives to the toxic antifungal and antibacterial wood preservatives that are still being used today, and the effect of microorganisms on oil-treated biocomposites has provided insight into the microstructural changes caused by their proliferation. Linseed oil inhibits the growth of the mold fungi *Rhizopus oryzae* and bacteria *Pseudomonas putida* on hemp-shivers (HS)- and corn starch (CS)-based biocomposites [13]. On the other hand, tung tree oil displayed fungal growth after 4 weeks and microbial growth after 6 months. Both oil films prevented microbial amylase activity on the CS biocomposites, but linseed oil-treated HS and CS biocomposites had lower cellulase activity and higher lipase activity than tung tree oil. The impregnation time of the oils before inoculation affected the protective coating’s efficacy over time, and the thermal conductivity and density of the impregnated biocomposites decreased for both oils. Linseed oil-treated biocomposites also had greater water absorption and a larger swelling thickness than tung tree oil biocomposites, which had higher efficacy as a water-resistant coating. The strength of the tung tree oil-treated biocomposites was greater after microbial incubation but ultimately they were not able to withstand degradation. Overall, linseed oil provided the biocomposites with better protection against microbial action.

The papers included in this Special Issue provide a glimpse into the current state of research on the microorganisms present in our environment. The vast communities of microorganisms across the globe allow us to advance our understanding of the world around us, mitigate the effects of the environmental damage we have caused as a species and explore the possibilities of improving and extending our lives through their applications in various branches of biotechnology.
